# ERAS® protocol improves survival after radical cystectomy: A single-center cohort study

**DOI:** 10.1097/MD.0000000000030258

**Published:** 2022-09-02

**Authors:** François Crettenand, Olivier M’Baya, Nuno Grilo, Massimo Valerio, Florence Dartiguenave, Yannick Cerantola, Beat Roth, Jean-Daniel Rouvé, Catherine Blanc, Ilaria Lucca

**Affiliations:** a Department of Urology, University Hospital CHUV, Lausanne, Switzerland; b Department of Anaesthesiology, University Hospital CHUV, Lausanne, Switzerland

**Keywords:** bladder cancer, enhanced recovery, radical cystectomy, survival

## Abstract

To evaluate Enhanced recovery after surgery (ERAS®) protocol on oncological outcomes for patients treated with radical cystectomy (RC) for urothelial carcinoma of the bladder (UCB).

**Methods:**

A prospectively maintained single-institutional database comprising 160 consecutive UCB patients who underwent open RC from 2012 to 2020 was analyzed. Patients receiving chemotherapy and those with a urinary diversion other than ileal conduit were excluded. Patients were divided into two groups according to the perioperative management (ERAS® and pre-ERAS®). The study aimed to evaluate the impact of the ERAS® protocol on survival at five years after surgery using a Kaplan–Meier log-rank test. A multivariable Cox proportional hazards model was used to identify prognostic factors for cancer-specific (CSS) and overall survival (OS).

**Results:**

Of the 107 patients considered for the final analysis, 74 (69%) were included in the ERAS® group. Median follow-up for patients alive at last follow-up was 28 months (interquartile range [IQR] 12–48). Five-years CSS rate was 74% for ERAS® patients, compared to 48% for the control population (*P* = 0.02), while 5-years OS was 31% higher in the ERAS® (67% vs. 36%, *P* = .003). In the multivariable analysis, ERAS® protocol and tumor stage were independent factors of CSS, while ERAS®, tumor stage so as total blood loss were independent factors for OS.

**Discussion:**

A dedicated ERAS® protocol for UCB patients treated with RC has a significant impact on survival. Reduction of stress after a major surgery and its potential improvement of perioperative patient’s immunity may explain these data.

## 1. Introduction

Despite tremendous improvement in surgical and anesthesiologic techniques over the years, radical cystectomy (RC) and bilateral pelvic lymph node dissection (PLND) for urothelial carcinoma of the bladder (UCB) remain highly morbid procedure.^[1]^ Patients diagnosed with muscle-invasive or treatment-refractory nonmuscle invasive UCB are those who benefit the most from this surgery. The 5-year disease-specific mortality rates for UCB are estimated to be as high as 30% to 50%,^[2,3]^ with a morbidity rate up to 70% at 30 days after RC.^[4]^

Enhanced recovery after surgery (ERAS®) is a multimodal protocol combining pre-, peri- and postoperative surgical, nutritional, and anesthesiologic elements. It has been designed to reduce surgical stress and standardize postoperative pathways.^[5]^ In UCB patients, an ERAS® protocol specific for RC has shown to significantly reduce morbidity, the length of stay (LOS), time to bowel recovery, and costs.^[6]^ In other malignancies, the adoption of ERAS recommendations has also been linked to improved overall and cancer-specific survival.^[7–9]^ Few data are, however, available on long-term benefits of ERAS® regarding oncological outcomes after RC.^[10,11]^ Indeed, as there is a reduction of surgical stress and better host immunity, one might argue that there might also be a positive impact of ERAS® on survival after RC and PLND^[12]^

The present study aimed to evaluate the impact of ERAS® on 5-year survival in patients treated with RC and PLND for UCB.

## 2. Material and methods

### 2.1. Patient selection

Following institutional review board approval (protocol number 2020-00919) we reviewed our prospectively maintained database comprising 160 consecutive open RC with bilateral PLND for muscle-invasive or treatment-refractory non-muscle invasive UCB between 2011 and 2018. Only UCB patients who had no neoadjuvant or adjuvant chemotherapy and those with an ileal incontinent urinary diversion were selected. Before ERAS® implementation, as required by the ERAS® society, a pre-ERAS® cohort was prospectively included as baseline sample in the same registry. Orthotopic neobladder reconstruction (N = 15), patients with postoperative follow-up time shorter than 3 months (N = 6), patients with extended nodal stage (pN2) (N = 20) and those with preoperatively known distant metastasis (N = 12) were also excluded.^[13]^ After applying exclusion criteria, 107 patients were included in the final analysis.

### 2.2. ERAS® protocol

Since 2012, all RC patients treated in our tertiary center have been managed according to ERAS® protocol as previously published.^[5]^ A multidisciplinary team (including urologists, clinical nurses, anesthesiologists, and physiotherapists) was created to continuously improve perioperative management based on ERAS® principles. The prospective ERAS® Interactive Audit System (EIAS) (Encare AB Stockholm, Sweden) registry was employed to prospectively collect preoperative data (age, gender, smoking status, body mass index, alcohol usage, nutritional status assessment, preoperative treatments, medical history, oral bowel preparation, antibiotic and thrombosis prophylaxis), intraoperative data (intraoperative blood loss, length of operation, type of anesthesia, IV volume, opioid use) and postoperative data (fluid balance, gastrointestinal function, mobilization, pain and nausea control, LOS, 30-days complication).

### 2.3. Data within the ERAS® database

Clinical data on patients and tumor characteristics, recovery parameters (pain score, bowel function, and mobilization), LOS, and 30-day complication rate were captured in EIAS by a dedicated nurse. The same parameters were collected retrospectively for the pre-ERAS® control group. Complications were graded according to the validated Clavien-Dindo classification. Grade I-II were defined as minor complications and grade III-IV as major complications. Tumors were staged according to the 2010 Tumor, Node, and Metastasis (TNM) classification. Tumor grade was re-assigned according to the 2004 World Health Organization grading system. For clinical staging, patients underwent multiphase contrast-enhanced computed tomography (CT) scans of the chest and abdomen. Main discharge criteria included adequate oral intake, adequate oral pain management, return of bowel function, sufficient mobilization, and stent free. No discharge was allowed if intravenous fluid therapy was still needed. All patients were examined by the surgeon-in-charge 1 month after discharge and interviewed by a dedicated ERAS® nurse to assess 30-day complication rates. Postoperative visits and follow-ups were scheduled at least every 6 months until the third year and annually thereafter according to European Association of Urology guidelines.^[14]^ The date and cause of death were obtained via regular and centralized follow-up.

### 2.4. Statistical analysis

Categorical variables are presented as numbers and proportions, and continuous variables by median and interquartile range (IQR). Adherence to the ERAS protocol was measured as the ratio of patients who were compliant with each item. Group differences in categorical variables and continuous variables were analyzed with chi-square tests and Mann–Whitney *U* tests, respectively. The Kaplan–Meier log-rank test was utilized to obtain and compare survival curves. A multivariable Cox proportional hazards model was fitted to identify independent, significant prognostic factors. Statistical testing was two-sided and a *P* value <.05 was considered statistically significant. Analyses were all conducted with STATA 16 (College Station, TX, USA).

## 3. Results

### 3.1. Patient’s characteristics

No differences were found in terms of demographic and oncological characteristics between the two groups (Table 1). Median follow-up was 28 months (IQR 12–48). Median LOS was 3 days shorter in the ERAS® group compared to the control population (15 days vs 18 days, *P* = .06). While minor complications were more frequent in the control cohort (87% versus 63%, *P* = .01), 30 days major complication rate was significantly higher in the ERAS® group (26 % versus 12 %, *P* = .01). The most common major complication were postoperative paralytic ileus (38 %, n = 28) and urinary tract infections (25 %, n = 18). Global adherence to ERAS® protocol was 67.3%

**Table 1:** Patient’s characteristic.

Variable	ERAS® group	Control group	*P* value
**N**	74	33	
Age - median (IQR)	72 (66–79)	71 (67–76)	.82
Gender-n (%)			.19
Female	25 (34.7)	7 (21.2)	
Male	49 (65.3)	26(78.8)	
Smoking-n (%)	23 (31)	9 (27.3)	.69
Diabetes-n (%)	19 (25.7)	4 (12.1)	.12
ASA class-n (%)			.72
I	1 (1.3)	0	
II	41 (55.4)	20 (60.6)	
III	32 (43.3)	13 (39.4)	
pT stage (%)			.06
pT0	5 (6.8)	5 (15.1)	
pTis	12 (16.2)	2 (6.1)	
pT1	13 (17.6)	3 (9.1)	
pT2	10 (13.5)	9 (27.3)	
pT3	27 (36.5)	6 (18.2)	
pT4	7 (9.4)	8 (24.2)	
pN stage-n (%)			.52
pN0	59 (79.7)	24 (72.7)	
pN1	14 (20.3)	9 (27.3)	
Follow-up - median (IQR)	23 (10–41)	36 (20–69)	.18
LOS - median (IQR)	15 (13–20)	18 (14–22)	.06
Complications-n (%)			.01
Minor	47 (63)	29 (87)	
Major	27 (36)	4 (12)	

### 3.2. Survival analysis

**Figure 1.**
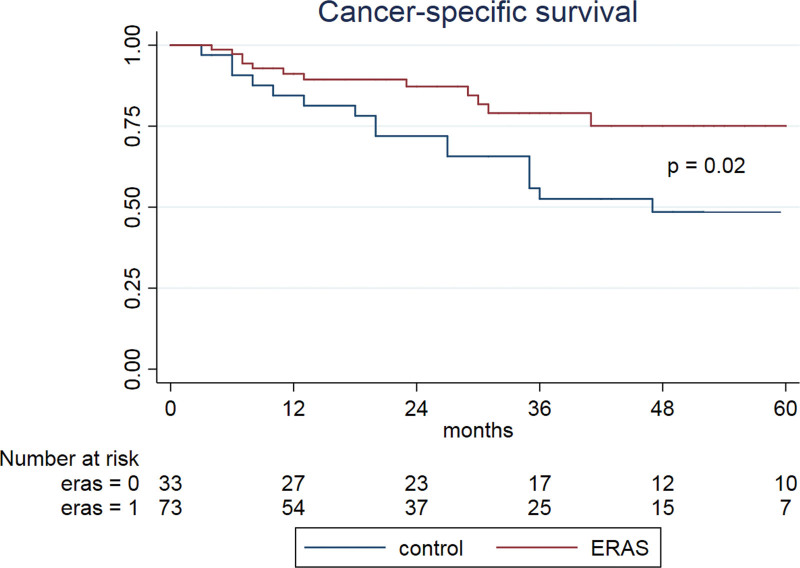
shows a statistically significant (*P* = .02) better cancer-specific survival (CSS), in patients included in ERAS® (enhanced recovery after surgery) group (red line) compared to standard of care (blue line) in pre-ERAS® era. Survival times is expressed in months and are calculated according to Kaplan-Meier estimator.

**Figure 2.**
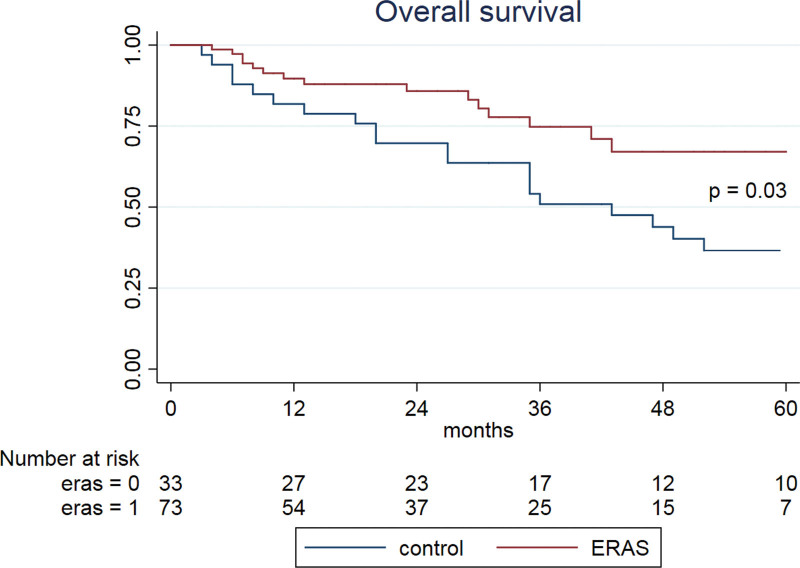
shows a statistically significant (*P* = .03) better overall survival (OS), in patients included in ERAS® (Enhanced recovery after surgery) group (red line) compared to standard of care (blue line) in pre-ERAS® era. Survival times is expressed in months and are calculated according to Kaplan–Meier estimator.

On a multivariable regression analysis, ERAS® protocol (Hazard Ratio [HR] 0.44; 95% CI 0.20–0.93, *P* = .01) and pathological tumor stage (HR 1.39; 95% CI 1.08–1.79, *P* = .01) were independent factors for cancer specific-survival (CSS) (Table 2), while ERAS® protocol (HR 0.4; 95% CI 0.19-0.80, *P* = .01), pathological tumor stage (pT) (HR 1.40; 95% CI 1.1–1.78, *P* = .005) and total blood loss (HR 1.01; 95% CI 1.00–1.08, *P* = .007) were found to be independent factors for Overall survival (OS) (Table 3). ERAS® patients had a 5-years CSS of 74% compared to 48% for the control population (*P* = .02, Fig. 1) and 5-years OS of 67 % compared to 36 % in the control group (*P* = .003, Fig. 2).

**Table 2:** Univariable and multivariable analysis for cancer-specific survival.

	Univariable			Multivariable		
Variable	HR	95% Cl	*P* value	HR	95% Cl	*P* value
Age	1.02	0.97–1.07	.44			
Gender	0.89	0.39–2.01	.07			
ASA Class	1.13	0.55–2.35	.12			
Smoking	0.77	0.34–1.83	.34			
Diabetes	1.26	0.54–2.96	.27			
Total blood loss	1.01	0.99–1.08	.2			
pN stage	1.28	1.00–1.64	.12			
pT stage	1.33	1.03–1.70	.02	1.39	1.08–1.79	.01
ERAS®	0.43	0.20–0.91	.02	0.44	0.2–0.93	.01

**Table 3:** Univariable and multivariable analysis for overall survival.

	Univariable			Multivariable		
Variable	HR	95% Cl	*P* value	HR	95% Cl	*P* value
Age	1.02	0.97–1.07	.27			
Gender	0.79	0.37–1.67	.53			
ASA Class	1.62	0.85–3.08	.14			
Smoking	0.70	0.32–1.54	.37			
Diabetes	1.17	0.54–2.60	.68			
pN stage	1.22	0.94–1.60	.12			
Total blood loss	1.01	1.00–1.08	.03	1.01	1.00–1.02	.007
pT stage	1.33	1.06–1.64	.01	1.40	1.1–1.78	.005
ERAS®	0.49	0.25–0.96	.04	0.4	0.19–0.80	.01

## 4. Discussion

In this retrospective single-center cohort study, we reported an association between ERAS® protocol and improved 5-year CSS and OS in UCB patients treated with RC. Both tumor pathological stage and ERAS® protocol were found independent predictors of survival.

Our results are consistent with those available for colorectal surgery, which is considered the pioneer in the ERAS® development. In a series of 911 patients undergoing major colorectal cancer surgery, a 42% reduction of cancer-specific mortality was found in those who had more than 70% adherence to ERAS® protocol.^[12]^ Similarly, improved OS was found with the use of minimal invasive surgery for colorectal cancer within an ERAS® setting.^[15,16]^

Very few studies have investigated the impact of ERAS® on RC survival.^[10,11]^ In a large cohort of 453 RC patients, Pang et al. found – in contrast to our data – similar cancer-specific and overall survival among ERAS® patients and the control group.^[17]^ However, the authors investigated mortality only at 30- and 90-days, but no long-term survival data are available. On the other hand, a prospective randomized study including 101 RC patients showed better CSS (58% vs. 49%) and OS rates in the ERAS® group compared to the conventional cohort, although the difference was not statistically significant.^[18]^ In both articles, no adherence to ERAS® guidelines was available. This a crucial information to define the final compliance with a possible impact on survival outcomes. In our series, adherence to ERAS® protocol was relatively high (67.3%), which was consistent with the published literature.^[19]^

Regardless of the type of surgery, no clear correlation or underlying mechanism has been found to explain the potential survival benefit of the ERAS® protocol. It is well-known that stress caused by major surgery and the postoperative healing process may reduce patients’ immunity, triggering residual disease and metastatic spread.^[20–22]^ Major pro-inflammatory cytokines and tumor growth promoters such as Interleukin 1, Interleukin 6 (IL-6), vascular endothelial growth factor (VEGF), transforming growth factor beta (TGF-β), and tumor necrosis factor-alpha (TNF-α) have been found increased during surgery.^[23]^ Furthermore, C-reactive protein (CRP), a well-known acute and chronic inflammatory marker mediated by IL-6 could promote tumor growth and spreading.^[24]^ Gakis et al. investigated the role of preoperative CRP levels on CSS among UCB patients treated with RC. Low CRP levels patients had a 3-year CSS of 74% compared to 44 % for those with a CRP level of more than 0.5 mg/dL.^[25]^ ERAS® protocol seems to reduce inflammatory cytokines, and increase immune function perioperatively, with a potentially positive effect on survival.^[26,27]^ Proposed strategies to reduce or at least minimize the stress caused by surgery are indeed a cornerstone of the enhanced recovery program.^[28]^

RC is considered a very invasive surgical procedure with one of the highest risks of complications among urological interventions. In our series, ERAS® patients had a higher rate of major complications at 30 days after RC compared to the control group, which is in contrast with most of the available data. The different collection of data among the two groups may explain these rates. The ERAS® database was prospectively and routinely completed by a dedicated study nurse attentive to report and score any deviation from standardized care-maps, whereas the pre-ERAS® data were retrospectively collected based on medical electronic records. In addition, no strict reporting guidelines were available and used for retrospective data collection in the retrospective cohort, as shown to alleviate potential bias.^[29]^ This suggests a measurement bias in favor of the retrospective pre-ERAS® cohort, as previously shown in the literature.^[30]^ Indeed, the 30-day major complication rate in the pre-ERAS group was only 12%, which is very low compared to the average reported in the literature.^[10,11]^ However, although the current literature^[6]^ indicates a reduction in complications, the ERAS® protocol is constantly evolving and the ideal regime may not yet be known. This study has limitations beyond the inherent shortcomings of any retrospective study including a possible selection bias and missing data. Both pre- and ERAS® patients were, however, collected prospectively following the EIAS registry, improving the quality of data. Furthermore, the study cohort was relatively small. However, the cohort was well selected based on strict inclusion criteria, excluding all chemotherapy treatment and other surgical variants other than ileal incontinent urinary diversion. As pre-ERAS® patients including those prior 2012, the median follow-up was slightly longer than ERAS® cohort, which may be a timing bias. However, no significant difference was found in the median follow-up between two populations. Another limitation was the lack of continuously conducted inflammatory blood tests such as CRP for pre-ERAS® patients, making the comparison with the ERAS® population impossible. Furthermore, the EIAS monitoring tool is not fully adapted to the specificity of RC. For example, the early removal of upper urinary tract drainage may lead to an increased risk of complications and is often postponed.^[19]^ The ERAS® protocol is built to evolve constantly, through routine audits of quality and analysis of collected data to challenge constantly the best practices. Therefore, the protocol was subject of continuous evolution over time. Our study suggests a positive impact of the ERAS® protocol on long-term RC survival and these data should motivate us to conduct a randomized, multicenter study, taking into account RC specificities. A dedicated ERAS® protocol for RC patients has a positive impact on CSS and OS. Nowadays impact of each proposed measure must be evaluated especially in terms of financial and cost effectiveness. For cystectomy patients, ERAS® allows cost savings ranging from 2800 to 4488 dollars per patient mostly driven by a shorter LOS and reduced need for intensive care unit.^[31]^ In addition, Hübner et al. showed a significant reduction of nursing workload associated with higher compliance to ERAS® protocol.^[32]^ Our data should prompt further implementation of enhanced recovery pathways in centers conducting RC in UCB patients. Large controlled studies investigating the potential mechanisms in an ERAS® setting are needed to better understand its benefit and to further improve global surgical care and disease survival in UCB patients undergoing RC.

## Author contributions

Conceptualization: François Crettenand, Olivier M’Baya, Yannick Cerantola, Beat Roth, Catherine Blanc, Ilaria Lucca.

Data curation: François Crettenand, Florence Dartiguenave, Massimo Valerio, Yannick Cerantola

Formal analysis: François Crettenand, Ilaria Lucca, Massimo Valerio, Nuno Grilo

Investigation: François Crettenand, Florence Dartiguenave

Methodology: François Crettenand, Ilaria Lucca, Massimo Valerio

Project administration: Francçois Crettenand, Yannick Cerantola, Jean-Daniel Rouvé

Supervision: Ilaria Lucca, Beat Roth, Catherine Blanc

Validation: François Crettenand, Ilaria Lucca, Beat Roth, Catherine Blanc

Visualization: François Crettenand, Ilaria Luca, Beat Roth

Writing – original draft: François Crettenand, Olivier M’Baya, Nuno Grilo, Jean-Daniel Rouvé

Writing – review & editing: Ilaria Lucca, Beat Roth, François Crettenand
